# Novel Kappa-Opioid Receptor Agonist for the Treatment of Cholestatic Pruritus: Systematic Review

**DOI:** 10.2196/30737

**Published:** 2022-05-02

**Authors:** Adrian Joseph Michel Bailey, Heidi Oi-Yee Li, Mark G Kirchhof

**Affiliations:** 1 Faculty of Medicine University of Ottawa Ottawa, ON Canada; 2 Division of Dermatology Department of Medicine The Ottawa Hospital Ottawa, ON Canada

**Keywords:** opioid, pruritus, cholestasis, dermatology, chronic pruritis, kappa-opioid receptor, opioid receptor

Chronic pruritus is a common and debilitating symptom associated with many dermatologic conditions and substantially impairs patients’ quality of life (QOL). In fact, the impact of chronic pruritis is thought to be comparable to that of chronic pain. Unfortunately, effective management for chronic pruritus remains limited and primarily consists of nonspecific measures, such as antihistamines and moisturizers.

There has been emerging evidence from various clinical trials demonstrating the efficacy and tolerability of a highly selective kappa-agonist, nalfurafine hydrochloride (TRK-820), for the treatment of pruritus in patients with chronic liver disease. Therefore, we conducted a systematic review to assess the efficacy of this agent in liver disease–associated pruritus.

PubMed and Embase were searched from inception to February 9, 2022, using the keywords “nalfurafine hydrochloride,” “itch,” and “pruritus” without restrictions. Two independent reviewers (authors AB and HOYL) screened and extracted data from all articles, with the supervising author (MK) providing consensus. All full-text single-arm, case-control, cohort, and randomized controlled trials with >10 patients describing the use of nalfurafine hydrochloride for the treatment of liver disease–associated pruritus were included. Editorials, commentaries, guidelines, and reviews were excluded. Outcomes included itch scores, QOL scores, and adverse events. The Cochrane Risk of Bias Tool 2.0 and the National Institutes of Health Pre-Post Study Quality Assessment Tool were applied to assess study quality (Multimedia Appendix 1).

Of 233 unique records, 5 studies were included (Figure 1). Study characteristics are summarized in Table 1. All studies were of low risk of bias or good quality.

**Figure 1:**
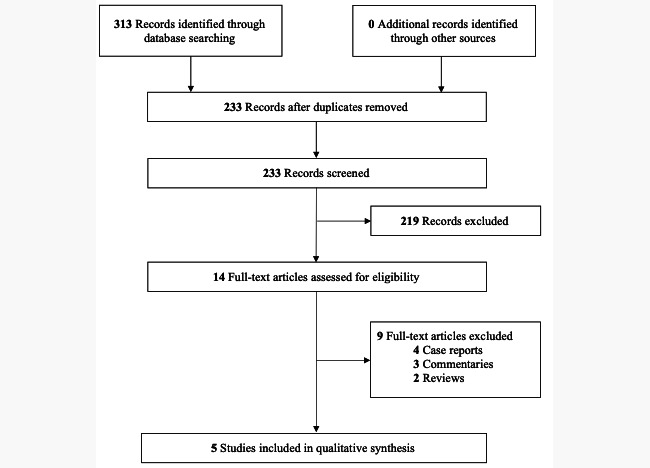
PRISMA (Preferred Reporting Items for Systematic Reviews and Meta-Analyses) flow diagram of the study selection process.

**Table 1:** Characteristics of included studies.

Study name	Country	Study type (data range)	Type of liver disease	Total participants, N (% female)	Age (years), mean	Treatment	Outcomes^a^	Study quality
Yagi et al, 2018 [1]	Japan	Single arm (2015-2016)	PBC^b^ with refractory pruritus	44 (89)	66.8 (SD 12.3)	2.5 mcg nalfurafine once daily for 12 weeks	VAS^c^: 42.9 at baseline to 29.3 at the end point (*P*=.001) PBC-40: 8.56 at baseline to 7.63 at the end point (*P*=.04)^d^ SF-36^e^: 42.9 at baseline to 29.3 at the end point (*P*=.001)^d^	Good
Akuta et al, 2018 [2]	Japan	Single arm (2015-2017)	Positive for HBsAg^f^ (n=19) Positive for HCV^g^ antibody (n=70) HCC^h^ (n=44) Others (n=5)	138 (53)	66 (range 24-91)	2.5 mcg nalfurafine once daily for a median of 6.4 (range 1-38) weeks	93 of 138 (67.4%) patients experienced a clinically relevant decrease in itch severity at the end point compared to baseline, predefined as a >50 mm decrease in their VAS score. This did not vary according to the etiology of liver disease (HBsAg+, HCV+, or HCC; *P*=.16).	Good
Yoshikawa et al, 2021 [3]	Japan	Single arm (2017-2018)	HCV (n=12) AFLD^i^ (n=5) NAFLD^j^ (n=1) PBC (n=5) Other (n=1) with refractory pruritus	24 (50)	68 (range 18-87)	2.5 mcg nalfurafine once daily for 12 weeks	17 of 24 (71%) patients experienced a clinically relevant decrease in itch severity at the end point compared to baseline, predefined as a >30 mm decrease in their VAS score. VAS: 50 at baseline to 25 at the end point (*P*=.001)	Good
Kumada et al, 2017 [4]	Japan	Randomized double-blind trial (2010-2012)	Chronic hepatitis (n=78) Cirrhosis (n=142) PBC (n=87) Others (n=28) with refractory pruritus	317 (57)	66.5 (SD 10.6)	2.5 mcg or 5 mcg nalfurafine once daily for 4 weeks	Decrease in VAS: 28.56 and 27.46 mm in the 2.5 μg and 5 μg groups at the end point from baseline, respectively, compared to 19.25 mm in the placebo group (*P*=.002 and *P*=.006, respectively)	Low risk of bias
Kamimura et al, 2018 [5]	Japan	Single arm (2015-2017)	PBC (n=11) AFLD (n=2) HCV (n=2) Vanishing bile duct syndrome (n=2) AIH^k^ (n=1)	11 (78)	69 (range 45-82)	2.5 mcg nalfurafine once daily for >20 weeks	The reduction in pruritus scores was correlated with the time of administration (Pearson correlation coefficient *r*^2^=0.636; *P*=.001).	Good

^a^Unless otherwise indicated comparisons between baseline and the end point across studies were determined using a paired Student *t* test for continuous and normally distributed variables and the Mann Whitney *U* test for variables without normal distribution.

^b^PBC: primary biliary cholangitis.

^c^VAS: visual analog scale.

^d^Both the SF-36 and PBC-40 are validated tools that assess the symptoms and health-related quality of life in patients with PBC.

^e^SF-36: 36-Item Short Form Health Survey.

^f^HBsAg: hepatitis B surface antigen.

^g^HCV: hepatitis C virus.

^h^HCC: hepatocellular carcinoma.

^i^AFLD: alcoholic fatty liver disease.

^j^NAFLD: nonalcoholic fatty liver disease.

^k^AIH: autoimmune hepatitis.

In a double-blind randomized controlled trial [4], patients with chronic liver disease and refractory pruritus experienced significant reductions in itch severity compared to a placebo capsule at 12 weeks, with a decrease in the visual analog scale of 41.6 and 39.3 mm in the 2.5 μg and 5 μg groups, respectively, compared to 32 mm in the placebo group (*P*=.007 and *P*=.03, respectively). The incidence of adverse drug reactions was higher in the experimental groups than in the placebo group. Patients reported these reactions were mild and did not impact patients’ daily activities. Major adverse drug reactions included polyuria, somnolence, insomnia, and constipation, all of which had a prevalence of 8% or lower at both doses and had a similar incidence in the placebo group.

Accounting for a combined 217 patients, 4 single-arm studies found that nalfurafine hydrochloride provided a clinically relevant decrease in itch severity in 67% to 71% of patients [2,3] and significantly improved patient QOL compared to baseline (PBC-40 decreased from 8.56 to 7.63, *P*=.04, and the 36-Item Short Form Health Survey decreased from 42.9 to 29.3, *P*=.001) [1], with no signs of dependence or abuse. The reduction in pruritus scores was also correlated with time of administration (*r*^2^=0.636; *P*=.001) [5].

In conclusion, nalfurafine hydrochloride has demonstrated efficacy in the treatment of liver disease–associated pruritis, significantly reducing itch scores compared to the placebo and improving patient QOL. Its advantage over nonspecific measures is its efficacy in refractory pruritus and favorable side effect profile. Considering this agent’s efficacy and tolerability, and the detrimental effect of refractory pruritus on patient well-being, dermatologists and other physicians should strongly consider this agent for future investigation and eventual use in chronic liver disease–associated pruritus.

## Appendix

Multimedia Appendix 1 Supplementary material.

## Abbreviations

QOL: quality of life
