# Supplementary material for: Novel Kappa-Opioid Receptor Agonist for the Treatment of Cholestatic Pruritus: Systematic Review
Source: JMIR Dermatol. 2022 May 2;5(2):e30737. doi: 10.2196/30737 (PMC10648544; doi:10.2196/30737)
Supplement: Multimedia Appendix 1 [file derma_v5i2e30737_app1.pdf]

Table S1. Search Strategy.

Database(s): **Embase Classic+Embase** 1947 to 2022 February 09**, Ovid MEDLINE(R) ALL** 1946 to February 09, 2022

Search Strategy:

#	Searches	Results
1	nalfurafine.mp. [mp=ti, ab, hw, tn, ot, dm, mf, dv, kf, fx, dq, nm, ox, px, rx, an, ui, sy]	499
2	itch.mp. [mp=ti, ab, hw, tn, ot, dm, mf, dv, kf, fx, dq, nm, ox, px, rx, an, ui, sy]	16495
3	pruritus.mp. [mp=ti, ab, hw, tn, ot, dm, mf, dv, kf, fx, dq, nm, ox, px, rx, an, ui, sy]	134615
4	2 or 3	141110
5	1 and 4	313
6	remove duplicates from 5	233

Table S2. Summary of the risk of bias assessments. N.B. The same rating was reached for all outcomes across studies. Green circles with a plus represent low risk of bias, yellow circles with a question mark represent some concerns for bias, and red circles with a minus represent high risk of bias. Full risk of bias assessments (i.e. answers to signaling questions) can be shared by contacting the corresponding author.

Study	Risk of Bias Arising from the Randomization Process	Risk of Bias due to Deviations from the Intended Interventions (effect of assignment to intervention)	Risk of Bias due to Deviations from the Intended Interventions (effect of adhering to intervention)	Risk of Bias due to Missing Outcome Data	Risk of Bias in Measurement of the Outcome	Risk of Bias in Selection of the Reported Result	Overall Risk of Bias
Kumada et al, 2017	+	+	+	+	+	+	+

Table S3. Scoring distribution of quality assessment of studies according to the National Heart, Lung, and Blood Institute Quality Assessment Tool for Case Series Studies (https://www.nhlbi.nih.gov/health-topics/study-quality-assessment-tools). Accessed October 2nd, 2021. Y = Yes, N = No, NR = Not Reported. Full quality assessments (i.e. answers to signaling questions) can be shared by contacting the corresponding author.

Study	Was the study question or objective clearly stated?	Was the study population clearly and fully described, including a case definition?	Were the cases consecutive?	Were the subjects comparable?	Was the intervention clearly described?	Were the outcome measures clearly defined, valid, reliable, and implemented consistently across all study participants?	Was the length of follow-up adequate?	Were the statistical methods well-described?	Were the results well-described?
Yagi et al, 2021	Y	Y	Y	Y	Y	Y	Y	Y	Y
Akuta et al, 2021	Y	Y	Y	Y	Y	Y	Y	Y	Y
Yoshikawa et al, 2021	Y	Y	Y	Y	Y	Y	Y	Y	Y
Kamimura et al, 2021	Y	Y	Y	Y	Y	Y	Y	Y	Y
